# Effect of myoglobin, hemin, and ferric iron on quality of chicken breast meat

**DOI:** 10.5713/ajas.20.0529

**Published:** 2020-11-09

**Authors:** Muhan Zhang, Weili Yan, Daoying Wang, Weimin Xu

**Affiliations:** 1 Institute of Agricultural Products Processing, Jiangsu Academy of Agricultural Sciences, Nanjing 210014, China

**Keywords:** Myoglobin, Hemin, Iron, Lipid Oxidation, Water Holding Capacity

## Abstract

**Objective:**

The objective was to evaluate the impact of different forms of iron including myoglobin, hemin, and ferric chloride on the quality of chicken breast meat.

**Methods:**

Chicken breast muscles were subjected to 1, 2, 3 mg/mL of FeCl_3_, myoglobin and hemin treatment respectively, and the production of reactive oxygen species (ROS) and malondialdehyde, meat color, tenderness, water holding capacity and morphology of meat was evaluated.

**Results:**

Hemin was found to produce more ROS and induce greater extent of lipid oxidation than myoglobin and ferric chloride. However, it showed that hemin could significantly increase the redness and decrease the lightness of the muscle. Hemin was also shown to be prominent in improving water holding capacity of meat, maintaining a relatively higher level of the immobilized water from low-field nuclear magnetic resonance measurements. Morphology observation by hematoxylin-eosin staining further confirmed the results that hemin preserved the integrity of the muscle.

**Conclusion:**

The results indicated that hemin may have economic benefit for the industry based on its advantage in improving water holding capacity and quality of meat.

## INTRODUCTION

Myoglobin (Mb), an essential hemoprotein in skeletal muscle, has been widely characterized for its functional role in oxygen storage and cellular diffusion. It consists of a globin moiety plus a porphyrin heme, the latter containing an iron atom that can exist in a reduced (ferrous) or oxidized (ferric) form coordinated inside the heme ring [1]. In meat, Mb is extensively studied because it plays a crucial role in meat colouration as the principle heme protein in sarcoplasm and because of its interaction with nitric oxide to form typical pigment in cured meat [2,3].

Moreover, lipid oxidation is the major factor that determines the sensory, functional, and nutritional quality of meat products, and Mb, released heme and iron has been recognized as major catalysts for lipid oxidation [4]. During post-mortem storage and processing of muscle, hemin (ferric heme) could dissociate from the globin, and heme oxygenase and reactive oxygen species (ROS) could cause heme destruction which subsequently releases iron from heme ring [3,5]. However, the relative contributions of different forms of iron, whether “free” or protein bound, heme or non-heme, in catalyzing lipid peroxidation in muscle-based foods have not been assigned. Min et al [6] reported that free ionic iron was more effective to promote lipid oxidation in raw and cooked chicken and beef meat. Whereas Grunwald and Richards [7] reported that released hemin is the primary promoter of lipid oxidation, and iron liberated from heme ring decreased the rate of lipid oxidation in washed fish muscle. The roles of myoglobin, hemin and free iron in lipid oxidation and their mechanisms is still not clear.

Despite their relevance to lipid oxidation, the contribution of these components to water holding capacity (WHC) and other meat quality traits is still an almost unexplored area. WHC is not only important for economic reasons as the industry loses money due to the weight loss of product and the inconsistency in the yield of the final product during storage or upon cooking, but also because of its role in the structure and sensory acceptability of meat. In this study, myoglobin, hemin and FeCl_3_, which represent protein-bound heme, free heme and free iron respectively were used to treat chicken breast muscle to investigate the efficiency of different iron forms in catalyzing lipid oxidation, and to assess their function in the color, tenderness and water retention of meat.

## MATERIALS AND METHODS

### Materials

Sixty-three white feather broiler chickens (fed for 40 days) were obtained from a commercial processing plant where they were slaughtered according to standard industry practices by hanging, electrical water bath stunning and bleeding from a unilateral neck cut severing the left carotid artery and jugular vein (Jiangsu Lihua Animal Husbandry Co., Ltd, Nanjing, China). Myoglobin (from equine skeletal muscle) and hemin were purchased from Sigma-Aldrich Corp. (St. Louis, MO, USA); All solvents and other reagents were analytical grade.

### Sample preparation

Two skinless, de-boned breast fillets (*Pectoralis major*) were taken immediately from each carcass and placed into a plastic bag on ice. The muscles were divided into 21 groups (n = 3), one of them was taken as fresh sample, two of them were immersed in phosphate buffered saline (PBS, pH 7.0) for 2 h and 8 h respectively, and another 18 groups were subjected to 1, 2, 3 mg/mL of FeCl3, myoglobin or hemin in PBS (pH 7.0) at 37°C in the dark for 2 h and 8 h respectively. The whole muscles were individually sealed in plastic bags, fully immersed in FeCl3, myoglobin, hemin or PBS solutions and were wiped with filter paper to remove excess liquid after the treatment. The measurements of meat quality were conducted immediately after the treatment, and the samples for other evaluation were frozen at −20°C.

### Reactive oxygen species detection

2,7-Dichlorofluorescein diacetate (DCFH-DA) was utilized as a chemical probe for ROS measurement [8]. Approximately 2 g of frozen muscles from each sample were crushed and homogenized on ice in 20 mL of Tris-HCl buffer (100 mM Tris-HCl, pH 8.0) and a protease inhibitors cocktail (Sigma-Aldrich Corp., USA) followed by centrifugation at 12,000 g for 10 min at 4°C. DCFH-DA was mixed with muscle extract at the final concentration of 10 μM, and they were incubated at 37°C for 30 min. The fluorescence intensity was measured at 485 and 525 nm as the respective excitation wavelength and emission wavelength on a Cytation5 microplate reader (Biotek Instruments Inc., Winooski, VT, USA).

### Determination of thiobarbituric acid reactive substances

Lipid oxidation of all samples was measured by the 2-thiobarbituric (TBA) method according to Sorensen and Jorgensen [9]. Ten grams of sample was homogenized with 30 mL of a 7.5% trichloroacetic acid solution containing 0.1% propylgallate and 0.1% ethylenediaminetetraacetic acid disodium salt for 30 s in an Ultra Turrax blender (9,500 rpm) and filtered through a Whatman filter No. 42. Equal 5 mL volumes of filtrate and 0.02 M TBA solution were mixed with glass stopped tubes and incubated in a water bath at 100°C for 40 min before cooling to room temperature under running cold tap water. The absorbance was measured at 532 nm using a spectrophotometer. Thiobarbituric acid reactive substances was calculated from a standard curve of malondialdehyde (MDA), freshly prepared by acidification of 1,1,3,3-tetraethoxypropane in the range from 0.02 μg/mL to 0.3 μg/mL and expressed as mg of MDA per kg sample.

### Meat color

The meat color (L*, a*, b*) was measured using a colorimeter (CR 400, Minolta, Tokyo, Japan). The colorimeter was calibrated using a standard white ceramic tile before measuring the samples.

### Cooking loss

Each sample was weighed accurately prior to cooking. The sample in cooking bag was immersed in an 80°C water bath until reaching an internal endpoint of 75°C. After cooking, the sample was cooled to the internal temperature of room temperature and wiped with blotting paper to remove excess water, followed by weighting immediately. Cooking loss was calculated as, Cooking loss (%) = ([raw weight − cooked weight]/raw weight)×100.

### Warner-Bratzler shear force

After measuring of cooking loss, the same muscle was then used for the determination of shear force. Breast portions were cut into 1×1 cm, variable length strips parallel to the muscle fibers. Shear force was determined through the application of the Meullenet-Owens razor shear test [10], using a texture analyzer (TVT-300XP, TexVol Instruments, Viken, Sweden) equipped with a razor blade with a height of 24 mm and a width of 8.9 mm. Muscle strips were cut across the fiber axis. The crosshead speed was set at 2 mm/s, and the test was triggered by a 10 g contact force. The shear was perpendicular to the axis of muscle fibers.

### Low-field nuclear magnetic resonance spin–spin relaxation (T_2_) measurements

Nuclear magnetic resonance (NMR) relaxation measurements were performed on a Niumag Benchtop Pulsed NMR Analyzer PQ001 (Niumag Electric Corporation, Shanghai, China) operating at a resonance frequency for protons of 22.6 MHz according to Bertram et al [11] with modifications. Approximately 2 g sample was placed in a 15 mm glass tube and inserted in the NMR probe. Spin-spin relaxation time, T_2_, was measured using the Carr-Purcell-Meiboom-Gill sequence. The T_2_ measurements were made with a τ-value (time between 90° and 180° pulse) of 200 μs Data from 12,000 echoes were acquired as 32 scan repetitions. The repetition time between subsequent scans was 6.5 s. Each measurement was performed in triplicate.

### Muscle specimen preparation and hematoxylin and eosin staining

Muscle tissues were sliced into 1 to 4 mm sections behind the optic chiasma and were subsequently fixed in 4% paraformaldehyde at 4°C according to Lan et al [12] with modifications. The sections were then dehydrated in a dehydration box. Melted paraffin was poured into the embedding box, followed by the muscle sections. When the paraffin was completely hardened, the paraffin block was removed from the embedding box and stored at 4°C. For slicing, the paraffin block was placed on a paraffin slicing machine and sectioned from the front to the back to make 4 μm thick sections. The slices, which floated on the surface, were flattened, and lifted onto the slides. The sections were then placed in a 60°C incubator overnight.

The sections were baked at 65°C for 30 min and then de-waxed twice in dimethylbenzene for 20 min each time. The sections were exposed to a gradient ethanol dehydration, and then hematoxylin (Baso Inc., Zhuhai, China) staining was performed for 5 min after the sections were washed in distilled water. The sections were then washed, immersed in 1% hydrochloric acid-alcohol solution, washed in water, treated with 0.6% ammonium hydroxide, washed again, and then stained with eosin (Baso Inc., China) for 1 to 3 min. Following gradient ethanol dehydration, the sections were dehydrated and cleared in dimethylbenzene. Gradient ethanol dehydration was performed again, and the sections were mounted in neutral balsam. Subsequently, morphological of the tissues were observed under a light microscope and photographed using a Nikon Eclipse Ci microscopic imaging system.

### Statistical analysis

All data were expressed as the mean±standard error. Statistical analysis of the differences between each group was evaluated at each time point by one-way analysis of variance using the SPSS 18.0 and values of p<0.05 was considered as statistically significant.

## RESULTS AND DISCUSSION

### Reactive oxygen species production and lipid oxidation

**Figure 1:**
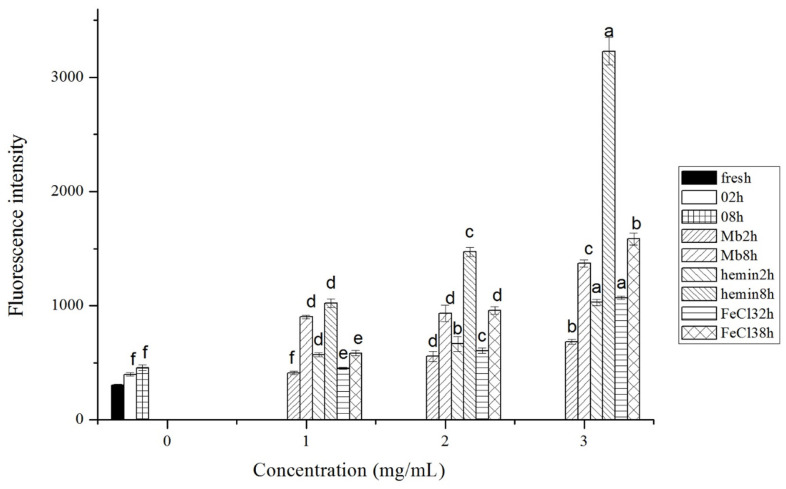
Reactive oxygen species (ROS) generation in fresh musles and muscles incubated with phosphate buffered saline (PBS) or different concentrations of myoglobin, hemin, and FeCl_3_ in PBS for 2 h or 8 h of incubation time, respectively. Results are expressed as mean±standard error. ^a–f^ For each incubation time, values not bearing common superscripts differ significantly (p<0.05).

The generation of ROS was examined via the DCFH-DA probe, which can be oxidized by ROS to form the fluorescent compound 2,7-dichlorofluorescein (DCF). As shown in Figure 1, the generation of ROS in chicken muscle increased with the concentration of Mb, hemin and FeCl_3_ and the incubation time. At the treatment time of 2 h, the level of ROS increased in 3 mg/mL hemin and FeCl_3_ treated muscle compared to that of Mb. While at 8 h, the level of ROS in hemin group exceeded the other two groups (p<0.05), and was in the order of hemin>FeCl_3_>Mb. ROS such as hydroxyl radical (·OH), superoxide anion (O_2_^−^), and hydrogen peroxide (H_2_O_2_) are highly reactive to initiate lipid peroxidation, protein fragmentation and DNA damage. The ferric ions are involved in the generation of hydroxyl and peroxide radicals in the presence of hydrogen peroxide known as Fenton reaction, or as catalysts in the Haber-Weiss reaction between H_2_O_2_ and superoxide to generate hydroxyl radicals [13]. Hemin can react with lipid hydroperoxides to form a wide array of lipophilic free radicals such as alkoxyl and peroxyl radicals [4,7]. During the autoxidation of Mb, superoxide anion (O_2_^−^) could be produced, and Mb has been shown to form the ferryl protein cation radical and transfer radicals to a wide range of target proteins [7,14]. Efforts have been made to determine the contributions of the different forms of iron to lipid oxidation in meat. The effects of heme pigments and non-heme iron on lipid oxidation in beef, pork and chicken were compared, and heme pigment concentration was found to be more important than non-heme iron in predicting lipid oxidation in meat [15]. The catalytic activity and the generation of radicals by metmyoglobin (metMb) and hemin have been studied experimentally and through computer simulation, and it showed that the activity of hemin exceeded that of the metMb at the physiological pH of 7.4, thus, the catalysis of lipid peroxidation by metMb is caused by the destruction of heme-protein adduct [16].

**Figure 2:**
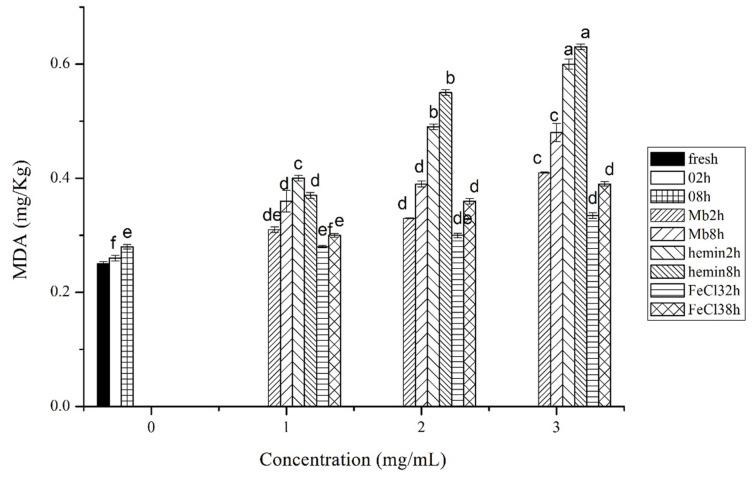
Malondialdehyde (MDA) formation in fresh musles and muscles incubated with phosphate buffered saline (PBS) or different concentrations of myoglobin, hemin, and FeCl_3_ in PBS for 2 h or 8 h of incubation time, respectively. Results are expressed as mean±standard error. ^a–f^ For each incubation time, values not bearing common superscripts differ significantly (p<0.05).

The production of MDA in chicken muscles showed that hemin could more effectively stimulate lipid oxidation than Mb and FeCl3 at each concentration and incubation time (Figure 2). The production of MDA was significantly higher in 3 mg/mL Mb incubated muscles than the same concentration of FeCl_3_ at 2 h and 8 h (p<0.05), but there was no significant difference between Mb and FeCl_3_ treated muscles at the concentration of 1 mg/mL and 2 mg/mL (p>0.05). The results coincided with the ROS measurements, indicating the formation of ROS is responsible for lipid oxidation in meat. The results could also be attributed to their different mechanisms in the reaction with lipids. Hemin is hydrophobic which enables its interaction with lipids, while Mb is hydrophilic and the interaction between Mb and lipids depends on the lipid concentration and the charge of the lipids [17]. In a high lipophilic environment denaturation of the protein occurs, which may result in heme release or further exposure of the heme group to the surrounding lipids [18]. As almost all lipids contain at least traces of peroxides, studies of lipid oxidation in aqueous colloidal systems suggest that the interaction between lipid hydroperoxides located at or near the droplet surface and transition metals occurs via radical chain reaction and is the most common cause of oxidative instability [19]. However, the rate of free iron induced lipid oxidation is determined by various factors such as the ratio of Fe^3+^/Fe^2+^, pH, the presence of chelators and other inhibitors etc. [20].

### Impact on meat color

Color is an important parameter that affects consumer decisions concerning the purchase of meat, Mb concentration and its state is accepted to be highly correlated with the color of the meat [3]. The meat in control group showed higher L* and lower a* values after 2 h and 8 h storage than the fresh meat (Table 1). Compared to the control, the meat by Mb treatment had a slightly lower L* and higher a* values. Hemin rendered meat a red color, with marked decrease in the lightness (L*) and significant increase in redness (a*) (p<0.05). However, the ferric chloride treatment led to the undesirable pale color and a reduction in redness. The resonant nature of the conjugated double bonds in the heme group is responsible for the ability of Mb to absorb visible light and function as a pigment [3]. The present results showed hemin could increase the redness of meat, but as chicken breast meat is considered as white meat, it might be more beneficial if it is applied to red meat. The possible explanation of the results is that association of hemin with lipid membranes may allow its penetration into the muscle cells [21], and hemin then reacted with H_2_O_2_ or lipid hydroperoxides to form ferryl intermediates of heme iron [22]. Furthermore, nitric oxide and carbon monoxide produced in muscle tissues could bind with hemin to form stable complexes to give bright red color [23].

### Impact on shear force of meat

**Figure 3:**
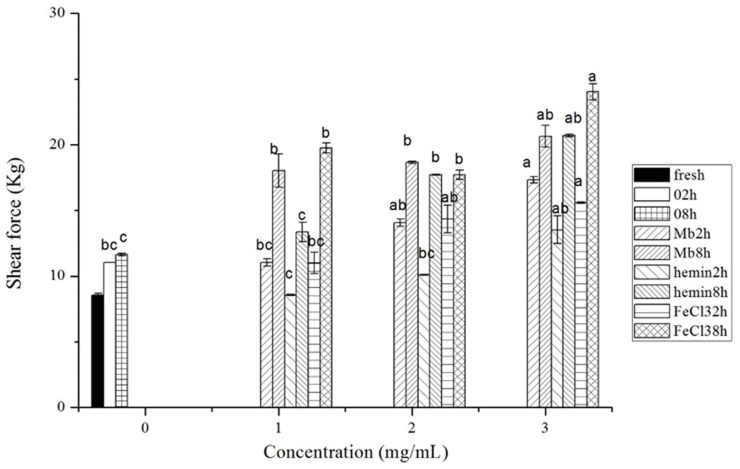
Shear force of fresh musles and muscles incubated with phosphate buffered saline (PBS) or different concentrations of myoglobin, hemin, and FeCl_3_ in PBS for 2 h or 8 h of incubation time, respectively. Results are expressed as mean±standard error. ^a–c^ For each incubation time, values not bearing common superscripts differ significantly (p<0.05).

As shown in Figure 3, there was no significant difference in tenderness of meat at the concentration of 1 mg/mL and incubation time of 2 h between control and each treatment (p>0.05). After incubated for 8 h, treatment of muscles with FeCl_3_ and Mb resulted in the significant decrease in tenderness (p<0.05), while tenderness of muscles treated with 1 mg/mL hemin had no marked change compared to that of the control (p>0.05). The shear force of meat by 1 mg/mL hemin treatment was comparatively low, but a further increase of the hemin concentration led to a decrease in tenderness. Physicochemical changes of structural proteins by proteases or attack by ROS and lipid oxidation by-products have been identified as contributing to the variation in meat tenderness [24]. Earlier studies have proposed that oxidation of myosin by hydroxyl radical generating systems (i.e. free iron and ascorbate) results primarily in disulfide bonds, whereas heme proteins cause the formation of di-tyrosine or other non-reducible cross-links [25]. Very low levels of oxidation could result in damage in myofibrillar proteins and activate proteasome, leading to degradation of structural proteins and may have merit in improving tenderness [26]. This may explain why low concentrations of hemin improved the tenderness. The decreased tenderness at higher concentration of these components might be attributed to the formation of cross-linkages between proteins, the loss of enzyme activity, the reduction in protein solubility, and limited proteolytic activity of proteases by oxidation [24].

### Impact on water holding capacity of meat

**Figure 4:**
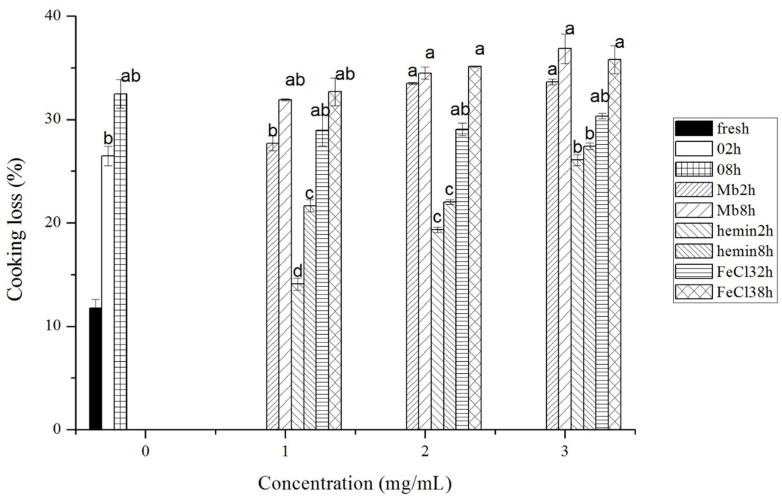
Cooking loss of fresh musles and muscles incubated with phosphate buffered saline (PBS) or different concentrations of myoglobin, hemin, and FeCl_3_ in PBS for 2 h or 8 h of incubation time, respectively. Results are expressed as mean±standard error. ^a–d^ For each incubation time, values not bearing common superscripts differ significantly (p<0.05).

Cooking loss of meat after different treatments is shown in Figure 4. With the increase of Mb, hemin and FeCl_3_ and treatment time, the cooking loss of meat increased. Mb treated meat had the highest cooking loss of up to 36.85% at the concentration of 3 mg/mL and incubation time of 8 h, while hemin treated meat had the lowest cooking loss. The cooking loss in 1 mg/mL and 2 mg/mL hemin treatment group was even significantly lower than that in control groups (p<0.05).

**Figure 5:**
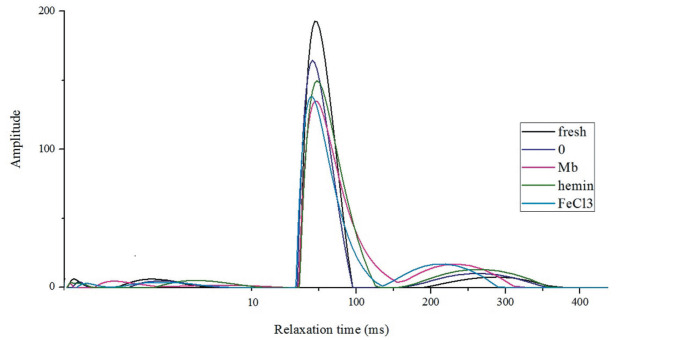
Representative distribution of T_2_ relaxation times for fresh muscles and muscles incubated with phosphate buffered saline (PBS) or 3 mg/mL myoglobin, hemin and FeCl_3_ respectively for 8 h.

Low-field NMR was used to determine the water distribution, water mobility and WHC of meat. Relaxation time T_21_, T_22_, and T_23_ at approximately 0 to 10 ms, 30 to 100 ms, and 200 to 300 ms are referred to as water that is tightly associated with macromolecules (bound water), located in myofibrillar network (immobilized water), and located outside the myofibrillar network (free water), respectively [27]. Immobilized water and free water accounted for more than 90% of total water. It can be noted from the curves that the main component T_22_ was highest in fresh meat, followed by the control group, and then the meat added with hemin (Figure 5). Mb and FeCl_3_ treated groups had much lower T_22_ peak area but higher T_23_ peak area, and T_22_ shifted to longer relaxation time while T_23_ shifted to shorter time, suggesting Mb and FeCl_3_ influenced the mobility of water. Most of the water affecting WHC of meat is immobilized water that is held either by steric effects or by attraction to the bound water [28]. After heating, some immobilized water become free water, which is implicated as cooking loss [27]. For the hemin treatment, the lower cooking loss was most probably due to the strong capacity of meat to retain the immobilized water, which accounted for a large proportion of all the water.

### Impact on morphology of meat

**Figure 6:**
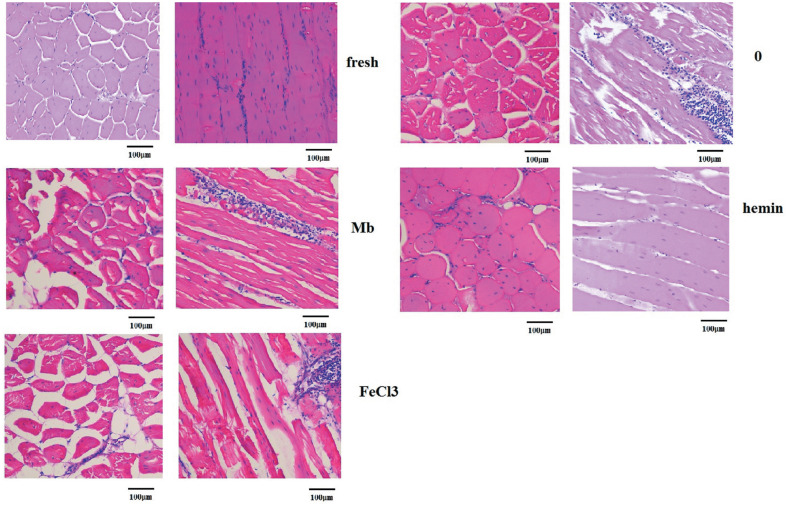
Representative images of hematoxylin and eosin staining of fresh muscles and muscles incubated with phosphate buffered saline or 3 mg/mL myoglobin, hemin and FeCl_3_ respectively for 8 h (×200).

The microstructure of meat samples was observed longitudinally and transversally with hematoxylin and eosin staining and light microscopy as shown in Figure 6. The microstructure of fresh meat samples was homogeneous and compact since the muscle fibers almost entirely filled the endomysial network and muscle bundles filled the perimysial network. Hemin treated samples displayed a similar morphology with only small spaces visible from the longitudinal view. The muscle fibers in the control group were arranged less compact in comparison with the fresh and hemin group. However, the muscle in Mb and FeCl_3_ treatment groups appeared very loose and severely damaged with more and larger drip channels along the muscle fiber direction. The fluid expelled due to myofibrillar shrinkage and accumulated in the extracellular spaces might be the source of water loss [29]. And increased proteolysis or oxidation of key cytoskeletal proteins such as the intermediate filament protein desmin was associated with reduced water loss [28]. It was hypothesized that lower level of desmin degradation resulted in the shrinkage of the muscle cells that translated into spaces between muscle cells and bundles, which ultimately lead to high drip loss or purge loss during postmortem storage [30]. In muscles, lipid oxidation and protein oxidation are always coexistent [31], the generation of ROS and lipid oxidation by-products by hemin may facilitate oxidation of these proteins and reduced the formation of drip channels and extracellular space. Furthermore, changes in cellular membranes and permeability have been recognized as influencing WHC of meat, and hemin could interact with cell membranes and influence the function, phase behavior and permeability of membranes [21]. This interaction may prompt the embedding of hemin in the membrane and allowing the access of hemin into cells to react with intracellular components. Since iron deficiency is the most prevalent micronutrient deficiency worldwide, and the bioavailability of heme iron is usually more efficient than non-heme iron [32], the treatment of meat with hemin could not only improve the WHC and quality of meat, but also provide a good source of iron.

## CONCLUSION

This study provides insights into the meat quality changes as influenced by myoglobin, hemin, and free iron. The results demonstrated that even though the addition hemin could result in higher levels of ROS production and lipid oxidation, it significantly improved the water holding capacity of meat and preserved the integrity of muscle. The results indicate that hemin may be an innovative substance to improve the quality of meat, and further studies will be needed to clarify how hemin participate in the structure changes and WHC of meat, as well as the transformation of different forms of iron in meat. Hemin, a natural component in meat, may have the potential to be used in the industry to minimize the water loss and improve meat quality.

## Figures and Tables

**Table 1:** Color of the meat incubated with different concentrations of myoglobin, hemin and FeCl_3_ respectively for different incubation time

Items		L*	a*	b*	L*	a*	b*
Fresh meat		47.59±0.36	3.71±0.12	2.54±0.19	-	-	-
		-------------------- 2 h of storage --------------------			-------------------- 8 h of storage --------------------		
0		58.96±0.39^b^	1.99±0.20^cd^	2.87±0.21^c^	62.82±0.75^bc^	1.59±0.07^d^	2.86±0.32^c^
1 mg/mL	Mb	56.13±1.60^bc^	1.32±0.34^d^	6.15±0.71^b^	58.62±0.85^c^	3.77±0.33^d^	5.52±0.44^b^
	Hemin	40.66±0.30^d^	4.07±0.60^b^	7.60±0.56^ab^	39.77±0.24^e^	5.19±0.30^c^	5.89±0.76^b^
	FeCl_3_	62.04±0.73^ab^	0.62±0.10^e^	3.16±0.46^c^	68.17±1.67^ab^	0.50±0.19^e^	4.35±0.36^b^
2 mg/mL	Mb	53.16±0.96^c^	2.65±0.43^c^	7.59±0.28^ab^	57.08±0.71^c^	4.36±0.42^cd^	7.06±0.48^ab^
	Hemin	33.65±3.09^e^	6.93±0.44^a^	8.55±0.68^ab^	35.00±1.40^f^	7.75±0.64^bc^	9.28±0.79^a^
	FeCl_3_	61.69±1.50^ab^	1.31±0.03^d^	4.31±0.34^bc^	64.37±0.94^b^	1.78±0.25^d^	4.88±0.28^b^
3 mg/mL	Mb	52.81±0.90^bc^	3.60±0.28^b^	9.06±0.75^a^	53.67±0.66^cd^	6.64±0.57^bc^	7.63±0.27^ab^
	Hemin	30.10±2.73^e^	7.57±1.10^a^	8.73±0.79^ab^	30.54±0.31^g^	11.77±0.78^a^	2.48±0.30^c^
	FeCl_3_	64.68±1.08^a^	0.58±0.05^e^	5.56±0.25^bc^	71.32±0.72^a^	0.97±0.28^de^	7.60±0.44^ab^

Results are expressed as mean±standard error.

a–g Values represented with different letters in the same column are statistically different (p<0.05).
